# DNA methylation-based lung adenocarcinoma subtypes can predict prognosis, recurrence, and immunotherapeutic implications

**DOI:** 10.18632/aging.104129

**Published:** 2020-11-21

**Authors:** Feng Xu, Lulu He, Xueqin Zhan, Jiexin Chen, Huan Xu, Xiaoling Huang, Yangyi Li, Xiaohe Zheng, Ling Lin, Yongsong Chen

**Affiliations:** 1 Department of Respiratory Medicine, The First Affiliated Hospital of Shantou University Medical College, Shantou, Guangdong, China; 2 State Key Laboratory for Diagnosis and Treatment of Infectious Diseases, The First Affiliated Hospital, College of Medicine, Zhejiang University, Hangzhou, Zhejiang, China; 3 Department of Pulmonology, Children's Hospital, Zhejiang University School of Medicine, Hangzhou, Zhejiang, China; 4 Department of Endocrinology, The First Affiliated Hospital of Shantou University Medical College, Shantou, Guangdong, China; 5 Department of Rheumatology, The First Affiliated Hospital of Shantou University Medical College, Shantou, Guangdong, China

**Keywords:** DNA methylation, lung adenocarcinoma, prognosis, recurrence, immunotherapy

## Abstract

The marked heterogeneity of lung adenocarcinoma (LUAD) makes its diagnosis and treatment difficult. In addition, the aberrant DNA methylation profile contributes to tumor heterogeneity and alters the immune response. We used DNA methylation array data from publicly available databases to establish a predictive model for LUAD prognosis. Thirty-three methylation sites were identified as specific prognostic biomarkers, independent of patients’ clinical characteristics. These methylation profiles were used to identify potential drug candidates and study the immune microenvironment of LUAD and response to immunotherapy. When compared with the high-risk group, the low-risk group had a lower recurrence rate and favorable prognosis. The tumor microenvironment differed between the two groups as reflected by the higher number of resting dendritic cells and a lower number of monocytes and resting mast cells in the low-risk group. Moreover, low-risk patients reported higher immune and stromal scores, lower tumor purity, and higher expression of *HLA* genes. Low-risk patients responded well to immunotherapy due to higher expression of immune checkpoint molecules and lower stemness index. Thus, our model predicted a favorable prognosis and increased overall survival for patients in the low-risk methylation group. Further, this model could provide potential drug targets to develop effective immunotherapies for LUAD.

## INTRODUCTION

Lung adenocarcinoma (LUAD), a common and aggressive subtype of non-small cell lung cancer (NSCLC), is the primary cause of cancer-related deaths worldwide [1–3]. The overall 5-year survival rate of patients with LUAD has remained low despite rapid advances in diagnostic techniques and molecular therapeutics [4]. Because molecular alterations in tumors occur earlier than clinical variations, novel and effective molecular biomarkers can accurately predict patient prognosis and cancer recurrence. Moreover, these biomarkers could be used to develop individualized treatment plans.

Epigenetic changes, such as DNA methylation, are inherited modifications that regulate gene expression, without any alteration in the underlying nucleotide sequence [5]. Aberrant DNA methylations are known to occur early during tumorigenesis in several cancers including LUAD [6, 7], and keep accumulating as cancer progresses [8]. Because different cancer subtypes display distinct methylation profiles [9–13], a DNA methylation-based model could provide an effective means to predict and identify potential cancer therapeutics.

DNA methylation is a process during which methyl groups are selectively added to CpG sites to form 5-methylcytosine [14]. We used the DNA methylation array data of LUAD from The Cancer Genome Atlas (TCGA) and Gene Expression Omnibus (GEO) databases to establish a robust prognosis and recurrence prediction model.

The tumor microenvironment of LUAD gets infiltrated by different immune cell types that contribute to malignancy [15–17]. Cancer immunotherapy involves the application of immune checkpoint blockers to stimulate the immune system against cancer cells. However, its beneficial effects have been reported in less than 20% of patients [18], and more reliable predictors of immune checkpoint blockade response are required. DNA methylation regulates the expression of several genes in the tumor microenvironment and could function as a reliable biomarker for these immune checkpoint blocks [19–22]. Publicly available drug sensitivity databases, including the Connectivity Map (CMap) at the Broad Institute and Genomics of Drug Sensitivity in Cancer (GDSC), could be used to identify candidate drugs against LUAD-specific DNA methylation signatures and develop individualized immunotherapy for patients with LUAD.

## RESULTS

### DNA methylation sites correlated with patients’ survival

The HumanMethylation 450K (HM450K) bead array data of 503 samples (471 LUAD and 32 normal lung tissue) were screened and 21,120 methylation sites were identified. After the exclusion of patients with no survival data, we studied the correlation between DNA methylation sites and patient prognosis with a univariate Cox regression analysis to assess the overall survival of patients with LUAD. Next, the penalized Cox analysis with the Least Absolute Shrinkage and Selection Operator (LASSO) was performed to narrow down the number of DNA methylation sites, which were selected 900 times over 1,000 repetitions. Finally, a stepwise multivariate Cox regression analysis was performed, and 16 methylation sites were identified as potential prognostic methylation sites. These were used to perform further analyses.

### Consensus clustering revealed distinct DNA methylation-based prognostic subgroups

**Figure 1:**
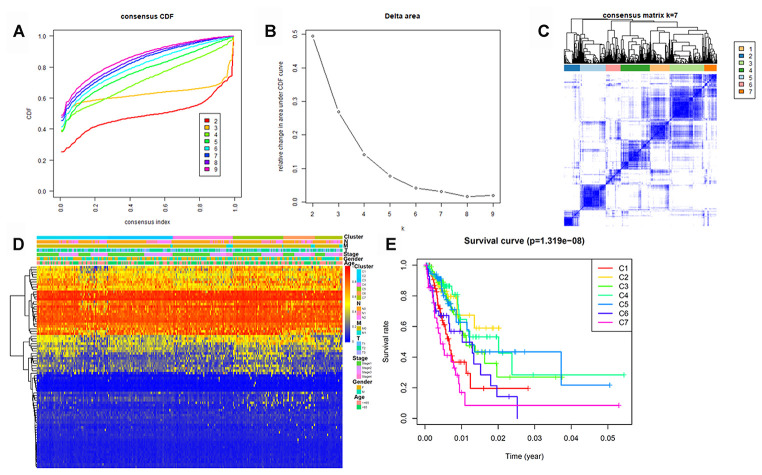
**Identification of DNA methylation-based clusters in LUAD samples.** (**A**) Consensus among DNA methylation-based clusters for each category number k. (**B**) Delta area curve of consensus clustering. (**C**) Consensus clustering of LUAD samples with k = 6. (**D**) Heatmap of LUAD methylation differences between each DNA methylation subtype. (**E**) Kaplan–Meier survival curves of LUAD in each DNA methylation subtype. LUAD, lung adenocarcinoma.

To determine DNA methylation-based clusters of LUAD, we performed an unsupervised hierarchical cluster analysis of patients with LUAD. Depending on the category number, the average cluster consensus and the coefficient of variation among clusters were calculated. We found that most samples in clusters 6 and 7 were stable (Figure 1A). Finally, the optimal cluster number assessed by the cumulative distribution function (CDF) delta area curve was 7 (Figure 1B). Therefore, we divided the samples into seven molecular subtypes. As shown in Figure 1C, a consensus matrix was used to identify the optimal number of clusters. The seven distinct clusters showed different DNA methylation profiles (Figure 1D). The Kaplan-Meier survival analysis revealed that DNA methylation affected the prognosis of patients with LUAD (Figure 1E).

### Generation and validation of the prognostic methylation model for patients with LUAD

**Figure 2:**
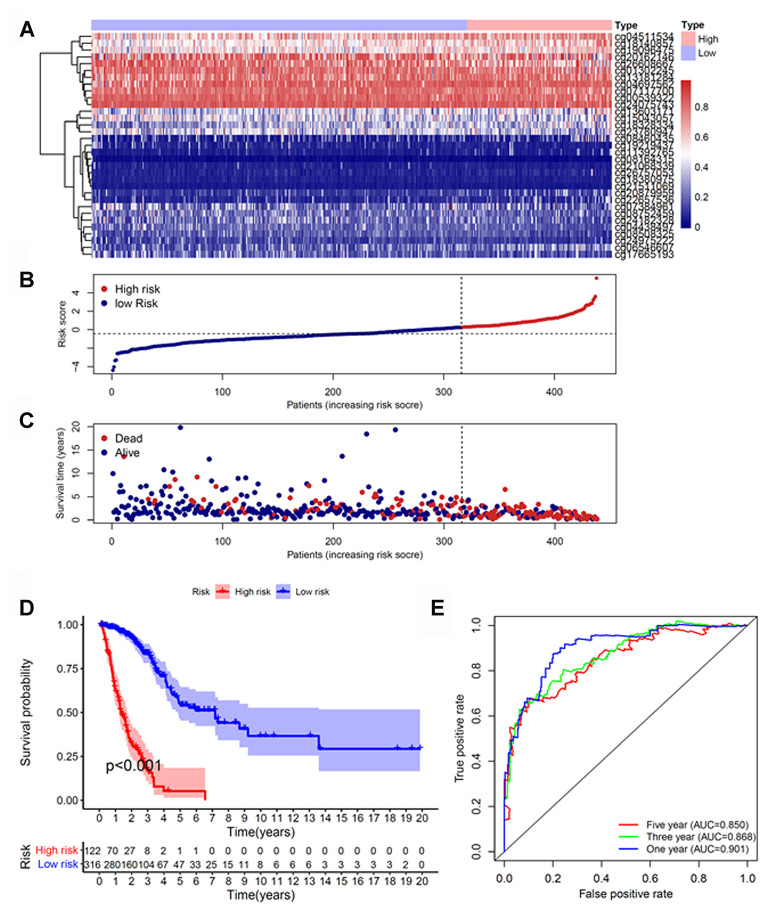
**Construction of the prognostic methylation model for patients with LUAD.** (**A**) The clustering analysis heatmap of methylation profile in DNA methylation signature sites. (**B**) The distribution of DNA methylation-based risk score. (**C**) Vital status of patients in the high- and low-risk groups. (**D**) Kaplan–Meier survival curves of the relative overall survival of patients in the high- and low-risk groups. (**E**) Accuracy of the prognostic model in predicting survival time by time-dependent ROC curve analysis. LUAD, lung adenocarcinoma; ROC, receiver operating characteristic.

**Figure 3:**
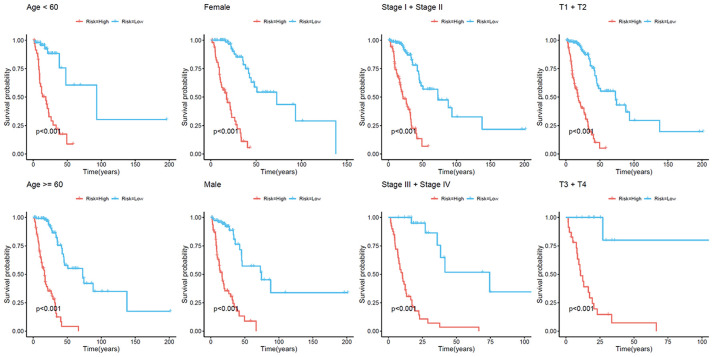
**Kaplan–Meier analysis of overall survival for patients with LUAD.** Patients were classified according to age (age < 60 and age ≥ 60 years), sex (male and female), TNM stage (Stage I + Stage II and Stage III + Stage IV), and T stage (T1 + T2 and T3 + T4). LUAD, lung adenocarcinoma; TNM, tumor/node/metastasis.

Because cluster 7 had the poorest prognosis and numerous CpG sites, we selected it as the seed cluster. DNA methylation profiles based on these 33 specific sites were measured for all samples, and we subsequently used them to calculate the risk score of each patient with LUAD. The optimal cut-off for dividing patients into high- or low-risk methylation group was set at 0.254 using the time-dependent receiver operating characteristic (ROC) curve analysis (Supplementary Figure 1). Figure 2A–2C display methylation profiles and risk score distribution. The Kaplan–Meier analysis showed that patients in the high-risk group had worse overall survival than those in the low-risk group (*p* < 0.001; Figure 2D). We next performed a ROC analysis to examine the specificity and sensitivity of the prognostic model. The time-dependent area under the curves (AUCs) for 1-, 3-, and 5-year overall survival rates of patients with LUAD using the prognostic model were 0.901, 0.868, and 0.850, respectively (Figure 2E). A higher AUC indicated better performance for LUAD-specific survival; thus, our data suggested excellent performance for survival prediction. To determine the reliability of the methylation model as a prognostic biomarker, patients with LUAD were stratified into different subgroups based on the following clinical characteristics: (1) age (age < 60 and age ≥ 60 years), (2) sex (male and female), (3) stage (Stage I + Stage II and Stage III + Stage IV), and (4) T stage (T1 + T2 and T3 + T4). Kaplan–Meier overall survival curves also showed that high-risk patients had shorter overall survival than low-risk patients in different subtyes, further indicating the excellent predictive ability of the methylation model (Figure 3).

Next, we validated the reliability and stability of the model using HM27K bead array data, with survival data in the external validation dataset. The risk scores of patients were calculated using the above-mentioned formula based on the optimal cut-off value. Patients with LUAD were subsequently classified into low- and high-risk groups (Supplementary Figure 2A–2C). Consistent with the above findings, patients in the high-risk group in the validation set had shorter overall survival than those in the low-risk group (*p* = 0.01; Supplementary Figure 2D). The AUC of the ROC analysis for the prognosis model was 0.824, implying high predictive accuracy and stability for survival prediction (Supplementary Figure 2E).

To study whether the model had similar prognostic values in different patients, we applied the same model to two other cohorts (GSE63384 and GSE83845) as external validation sets. The risk score for each patient with LUAD was calculated using these 33 CpG methylation sites. The patients were assigned to low- and high-risk groups (Supplementary Figure 3A–3C). Patients in the high-risk group had poorer overall survival in the meta-GEO dataset than those in the low-risk group (Supplementary Figure 3D). The AUC of the ROC analysis was 0.650 (Supplementary Figure 3E).

For further internal validation of the methylation model, patients with LUAD from TCGA HM450K were randomly divided into training and testing sets. Supplementary Table 1 shows the baseline characteristics of these two sets. No significant differences in clinical properties were observed between the two datasets (*p* > 0.05). We used the same risk score formula and computed the risk score for all patients in the training and testing cohorts (Supplementary Figure 4A–4C and 4F-4H). In line with the findings of the TCGA and meta-GEO cohorts, high-risk patients had shorter overall survival than low-risk patients in both cohorts (Supplementary Figure 4D and 4I). Time-dependent ROC analysis indicated that the AUCs for 1-, 3-, and 5-year overall survival rates in the training cohort were 0.895, 0.874, and 0.895, respectively (Supplementary Figure 4E). Moreover, the risk score-based classification of the testing TCGA cohort yielded similar results. The AUCs for 1-, 3-, and 5-year overall survival rates were 0.906, 0.865, and 0.819, respectively (Supplementary Figure 4J).

### Prognostic methylation profiles function as a recurrence model for patients with LUAD

**Figure 4:**
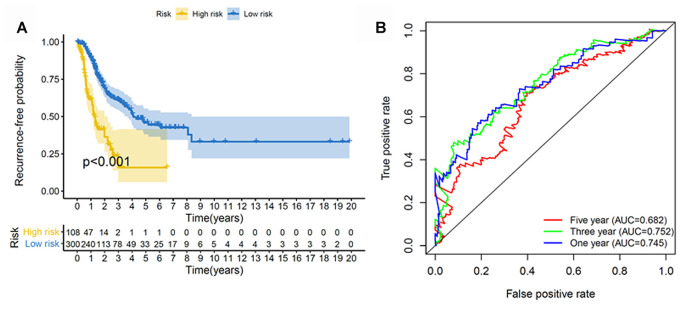
**Construction of the recurrence methylation model for patients with LUAD.** (**A**) Kaplan-Meier curves of the recurrence model of patients in the high- and low-risk groups. (**B**) Accuracy of the prognostic model in predicting recurrence rate by time-dependent ROC curve analysis. LUAD, lung adenocarcinoma; ROC, receiver operating characteristic.

**Figure 5:**
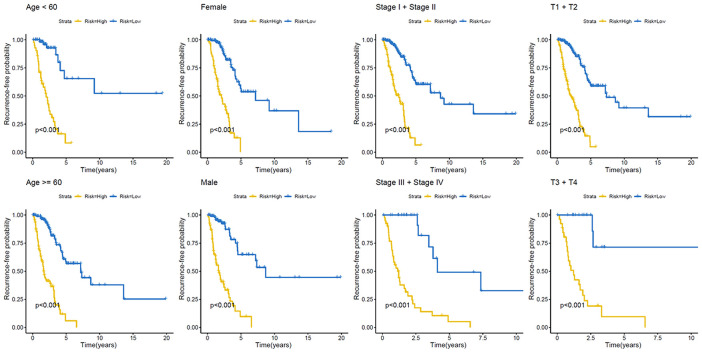
**Kaplan–Meier analysis of recurrence-free survival of patients with LUAD.** Patients were classified according to age (age < 60 and age ≥ 60 years), sex (male and female), TNM stage (Stage I + Stage II and Stage III + Stage IV), and T stage (T1 + T2 and T3 + T4). LUAD, lung adenocarcinoma; TNM, tumor/node/metastasis.

We next constructed a recurrence model using specific methylation sites and disease-free survival time and recurrence status in the dataset from HM450K bead array. When compared with patients in the low-risk group, those in the high-risk group had an elevated recurrence rate (*p* < 0.001) (Figure 4A). The recurrence model resulted in an AUC of 0.682, 0.752, and 0.745 for 1-, 3-, and 5-year disease-free survival time, respectively (Figure 4B). These results verified the predictive accuracy of the recurrence model. Further, these results validated the moderate sensitivity and specificity of the prognostic model. Next, we used this recurrence model to stratify patients with LUAD based on their clinical characteristics such as age, sex, stage, and T stage subgroups (Figure 5).

### Tumor immune microenvironment of patients with high- and low-risk LUAD

**Figure 6:**
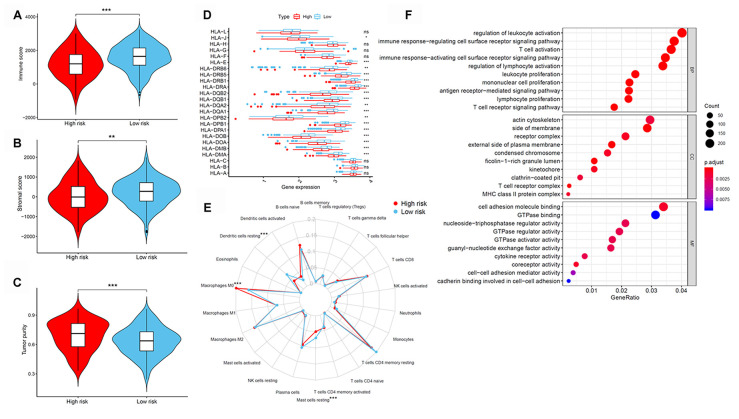
**Tumor immune microenvironment of patients in high- and low-risk groups with LUAD.** (**A**) Immune scores. (**B**) Stromal scores. (**C**) Tumor purity between patients with high and low risk. (**D**) The expression of *HLA* genes between patients with high and low risk. (**E**) The difference in immune cell infiltration in different LUAD subtypes. (**F**) GO enrichment analyses. GO, gene ontology; HLA, human leukocyte antigen; LUAD, lung adenocarcinoma.

Next, differences in immune cell infiltration of tumor microenvironment were studied in patients with high- and low-risk LUAD. The Estimation of STromal and Immune cells in MAlignant Tumor tissues using Expression data (ESTIMATE) algorithm revealed that the immune and stromal scores were higher in patients in the low-risk group than those in the high-risk group (Figure 6A and 6B). Moreover, we compared the tumor purity of the three LUAD subtypes and found opposite trends—patients in the high-risk group ranked higher than those in the low-risk group (Figure 6C). Because of their clinical implications in immunotherapy, we investigated any potential correlation between the LUAD subtypes and the expression of human leukocyte antigen (*HLA*) genes. Interestingly, patients in the low-risk group reported higher expression of the majority of *HLA* genes than those in the low-risk group (Figure 6D). We next evaluated the differences in immune infiltration of 22 immune cell types between the two groups using the CIBERSORT method in association with the LM22 model matrix. Compared with the high-risk group, patients in the low-risk group had a higher number of resting dendritic cells and a lower number of monocytes and resting mast cells (Figure 6E). To study the biological characteristics of differentially expressed genes between high- and low-risk patients with LUAD, we conducted gene ontology (GO) enrichment analyses. We found differentially expressed genes to be clustered and mostly enriched in immune functions, such as antigen receptor-mediated signaling pathways, immune response-regulating cell surface receptor signaling pathways, immune response-activating cell surface receptor signaling pathways, regulation of lymphocyte activation, and T cell activation (Figure 6F).

### Immunotherapeutic response of LUAD subtypes

**Figure 7:**
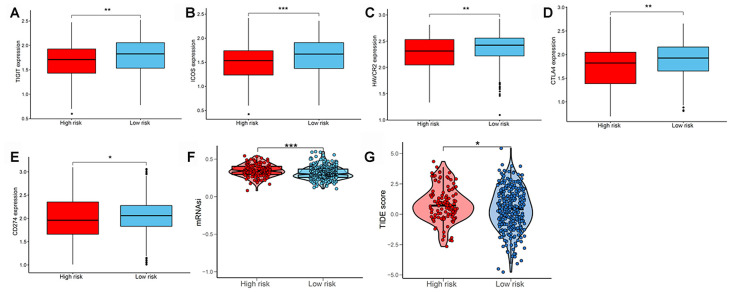
**Evaluating the immunotherapeutic response of LUAD subtypes.** The expression of immune checkpoint molecules including (**A**) TIGIT, (**B**) ICOS, (**C**) TIM-3 (HAVCR2), (**D**) CTLA4, and (**E**) PD-L1 (CD274) between patients with high and low risk. (**F**) Stemness index values of patients in high- and low-risk groups. (**G**) Immunotherapeutic responses of patients with high and low risk. LUAD, lung adenocarcinoma; PD-L1, programmed cell death-ligand 1.

We subsequently determined the expression of several key immunomodulators, including TIGIT, ICOS, TIM-3 (HAVCR2), CTLA4, and PD-L1 (CD274) to study the immunotherapeutic response. As shown in Figure 7A–7E, low-risk patients had higher expression of immune checkpoint molecules than high-risk patients. Cancer stem cells are vital for cancer growth, metastasis, and recurrence, and contribute to the resistance of tumors to conventional radiation therapy and chemotherapy. We used a one-class logistic regression (OCLR) machine-learning algorithm to calculate the tumor mRNA expression-based stemness index (mRNAsi). We observed that patients with the high-risk LUAD subtype had elevated stemness indices compared with those in the low-risk group (Figure 7F). Next, we used the Tumor Immune Dysfunction and Exclusion (TIDE) algorithm to predict the likelihood of the response to immunotherapy. Interestingly, we found that patients in the low-risk group were more likely to respond to immunotherapy than those in the high-risk group (Figure 7G). These data further supported our finding that patients with low-risk LUAD subtype had better prognosis and might respond well to immunotherapies.

### Identification of novel candidate drugs targeting the methylation signature

**Figure 8:**
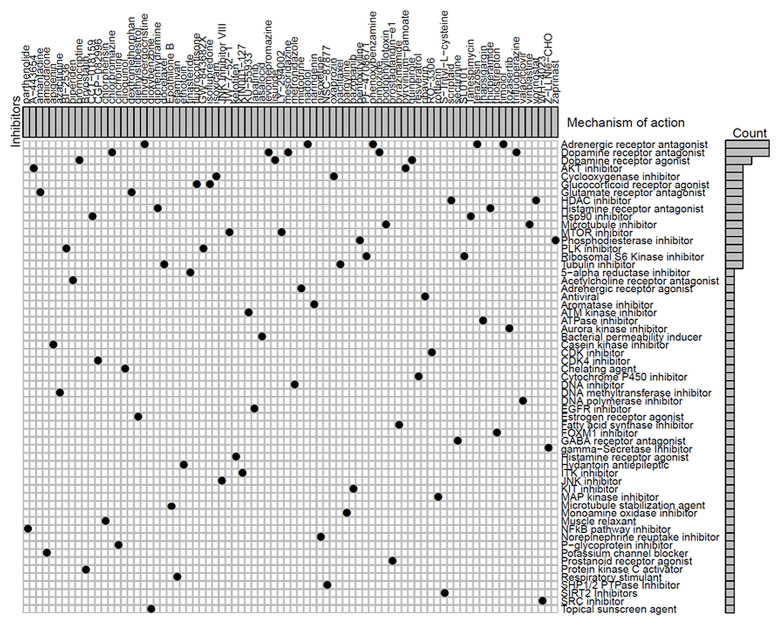
**Identification of novel candidate drugs targeting methylation signatures.**

We next identified 82 compounds as potential drugs targeting the methylation signatures. The mode-of-action (MoA) analysis of these compounds revealed 59 shared mechanisms of action (Figure 8). The analysis using predictive databases, i.e., CMap and GDSC revealed that five drugs (phenoxybenzamine, terazosin, timolol, dihydroergocristine, and nadolol) shared the MoA of an adrenergic receptor antagonist, five drugs (levomepromazine, trifluoperazine, chlorpromazine, mesoridazine, and pimozide) shared the MoA of a dopamine receptor antagonist, and three drugs (lisuride, quinpirole, and bromocriptine) shared the MoA of a dopamine receptor agonist. Thus, we identified drugs that targeted different methylation profiles in patients with LUAD and could be used for further analysis.

### Correlations between the methylation model and clinical properties

**Figure 9:**
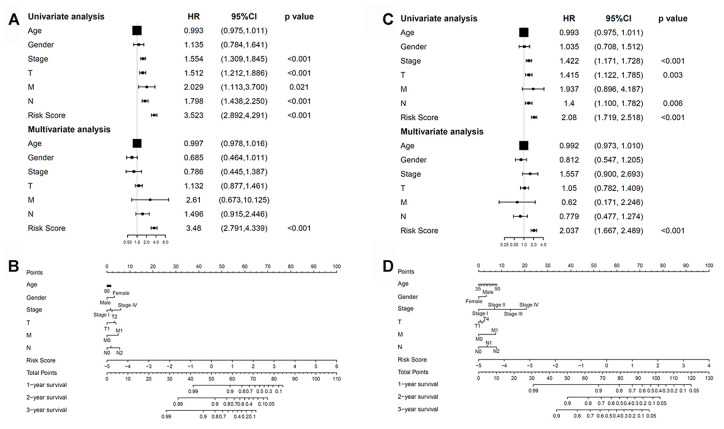
**Correlations between the methylation model and clinical characteristics.** (**A**) The prognostic model. (**B**) Nomogram for predicting the probability of 1-, 3-, and 5-year overall survival of patients with LUAD. (**C**) The recurrent model. (**D**) Nomogram for predicting the probability of 1-, 3-, and 5-year disease-free survival of patients with LUAD. LUAD, lung adenocarcinoma.

We next checked whether the prognostic model was independent of other conventional clinical properties. Univariate Cox regression analysis revealed that the tumor/node/metastasis (TNM), T, M, and N stages and the risk score correlated with poor survival. Multivariate Cox regression analysis revealed risk score as a specific prognostic indicator for LUAD (*p* < 0.001; Figure 9A). Subsequently, a nomogram integrating the seven factors was constructed for predicting 1-, 3- and 5-year overall survival rates. Compared with the clinical properties, the risk score for the prognostic model displayed superior predictive performance in the nomogram (Figure 9B).

Univariate and multivariate Cox regression analyses were further performed to examine whether the recurrence model was independent of other clinical properties. Cox regression analyses revealed the recurrence model to be significant (*p* < 0.001; Figure 9C). Next, a nomogram that integrated the risk score and clinical risk factors was constructed, in which the risk score for the recurrence model demonstrated good accuracy for predicting 1-, 3- and 5-year disease-free survival rates of patients with LUAD (Figure 9D).

## DISCUSSION

Over the past several decades, LUAD has become a major public health concern due to its highly malignant nature [23]. The tumor microenvironment is known to contribute to tumorigenesis and malignant phenotypes [15, 16]. Furthermore, DNA methylation contributes to tumorigenesis by altering the tumor microenvironment of several cancers including LUAD. For example, aberrant DNA methylation can alter immune functions such as T cell differentiation and T cell exhaustion, and the expression of inhibitory immune checkpoint genes, such as *PD-L1*, *PD-L2*, and *CTLA4* [24].

Immune checkpoint blockade or immunotherapy is a promising strategy for treating various cancers. For example, antibodies against programmed cell death-1/programmed cell death-ligand 1 (PD-1/PD-L1) immune checkpoint pathway rescued the tumoricidal function of effector T cells [25]. Similarly, anti-PD-1 antibodies are effective in treating several cancers including LUAD, and improving the overall survival [26–28]. However, not all patients with lung cancer respond well to these inhibitors, which could be attributed to checkpoint inhibitor complexity and patients’ limited tumor immunity [29, 30]. An improved immune signature-based classification of LUAD could identify subsets of patients who may benefit the most from current therapies [31]. For instance, Xue et al. verified that DNA methylation signatures could reliably predict the immunotherapy response and function as effective biomarkers [19].

We identified 33 DNA methylation sites as novel prognostic and recurrence biomarkers and therapeutic targets for LUAD. Based on clinical characteristics, such as age, sex, TNM stage, and T stage, patients were divided into subgroups to validate the independent predictive value of methylation signatures and study the difference in the overall survival and recurrence rate between the high- and low-risk methylation groups.

Cancer stemness is associated with worse outcomes and suppressed immune responses, such as reduced expression of *PD-L1* [32]. We found that patients with low-risk LUAD subtype reported higher immune and stromal scores, infiltration of resting dendritic cells, and elevated expression of *HLA* and immune checkpoint genes. Moreover, the mRNAsi negatively correlated with the LUAD methylation level, suggesting that DNA methylation negatively affected the transcriptome of LUAD stem cells.

The DNA methylation model could effectively predict the overall survival and recurrence rates, independent of patients’ clinical properties. Genes predicted by this model were specifically enriched in immune response. Prediction using the TIDE algorithm indicated that patients with low-risk subtype responded well to immunotherapy. Based on these results, we speculate that this prediction model could provide reliable immune biomarkers for cancer therapy. Further, using CMap and GDSC databases, we identified 82 potential compounds with 59 MoAs and higher immune response in the low-risk group; these could be used to target DNA methylation signatures to treat patients with LUAD.

Dendritic cells express adrenergic receptors on their surfaces, stimulation of which by β-agonists modifies the cytokine secretion profiles of these cells [33]. Dendritic cells express dopamine receptors in addition to the machinery necessary to synthesize, store, and degrade dopamine [34]. Glutamate, released by Dendritic cells, is a novel and highly effective regulator in the initiation of T cell-mediated immune responses during T cell–DC interaction [35]. The role of histone deacetylases (HDACs) in the epigenetic regulation of innate and adaptive immunity is of significant interest. HDAC inhibition acetylated and activated signal transducer and activator of transcription-3 (STAT-3), which was critical for the induction of indoleamine 2,3-dioxygenase (IDO) and regulation of Dendritic cells [36]. The phosphatidylinositol-3 kinase/protein kinase B/mammalian target of rapamycin (PI3K-Akt-mTOR) pathway is an important upstream regulator of glycolytic metabolism and plays a central role in Dendritic cells activation and immune responses [37]. Heat shock protein 90 (Hsp90) plays a critical role in protein folding, transport, and cellular activity. Hsp90 was shown to inhibition significantly inhibit Dendritic cell function [38].

During routine clinical work, the pathological stage functions as a prognostic determinant for lung cancer. However, patients with LUAD in the same stage report different clinical outcomes, revealing the inaccuracy of current staging systems in making reliable predictions and revealing LUAD heterogeneity. Therefore, it is necessary to obtain potential predictive and therapeutic biomarkers. The established DNA methylation model offers a novel method for identifying patients with LUAD in addition to predicting the prognosis and recurrence and taking therapeutic decisions.

## MATERIALS AND METHODS

### Data source and pre-processing

The DNA methylation data of patients with LUAD were generated by Illumina Infinium HumanMethylation 450K and 27K BeadChips (HM450K and HM27K) using the UCSC genome browser. Methylation levels of each CpG site were expressed using the β-value that ranged from unmethylated to fully methylated. First, we excluded the CpG sites with a missing ratio of more than 70% of all samples. Next, we excluded cross-reactive genome CpG sites based on the identification of cross-reactive probes and polymorphic CpGs in the Illumina Infinium HumanMethylation microarray [39]. In addition, the CpG sites in the sex chromosomes were excluded, and those in the promotor regions were further examined [40]. In total, 503 samples and 21,120 methylation sites for HM450K, and 150 samples and 21,120 methylation sites for HM27K were included in subsequent analyses. RNA sequencing and its clinical LUAD patient data were obtained from the TCGA website. A univariate Cox regression analysis was performed to determine the association between the methylation level of each CpG site and the overall survival of patients with LUAD for HM450K and to identify CpG sites related to overall survival (*p*-value < 0.05). After primary filtration, a LASSO Cox regression analysis was performed to reduce the number of CpG sites using the R package “glmnet”. A multivariate Cox regression analysis was ultimately performed to evaluate the contribution of CpG sites as an independent predictive indicator for patients with LUAD, as previously described [41, 42].

### Consensus cluster analysis to identify methylation-based subtypes

To perform the consensus classification of LUAD for HM450K, we used the R package “Consensus ClusterPlus”, which provides stable quantitative and visual evidence for estimating the number of unsupervised clusters in a dataset [43]. In each cluster, 80% of the tumors were sampled 100 times, and a k-means algorithm with the Euclidean metric was used. The clustering number was assessed according to the area under the CDF curve [39].

### Construction and validation of a prediction methylated risk model

The above-mentioned specific methylation sites were used to construct a prediction model. To validate the methylated signature, the risk score was calculated according to the prognostic signatures in two GEO datasets (GSE63384 and GSE83845) using the R software package “GEOquery”. Next, patients with LUAD from TCGA were randomly divided into training and testing cohorts as additional validation datasets.

### Evaluation of immune microenvironment

Immune scores were evaluated by applying the ESTIMATE algorithm to the gene expression data from TCGA [44, 45]. Tumor purity was obtained based on the ESTIMATE score using a fitted formula as previously described [45].

### Estimation of tumor-infiltrating immune cells

The normalized gene expression data with standard annotation files were uploaded to the CIBERSORT web portal. Next, the algorithm was determined by 1,000 permutations and LM22 gene signature, as described in previous studies [42, 46]. The R “Genefilter” package was used to screen each LUAD sample, and a *p*-value < 0.05 was used to set the threshold.

### Functional enrichment analysis

The GO analysis was performed on differentially expressed genes using the R “clusterProfiler” package [47]. The thresholds for analyses were set using a *p*-value < 0.05 that indicated enriched functional annotations.

### Calculation of stemness index

Stemness indices were calculated using an innovative OCLR machine-learning algorithm, as previously described [44, 48]. Next, we calculated Spearman’s correlations between the stemness index model and the lung cancer sample expression profiles based on the data obtained from TCGA. The stemness index was mapped to the [0,1] range using a linear transformation that subtracted the minimum and divided the maximum value.

### Immunotherapeutic response prediction

Several immune checkpoint pathways are involved in tumor immune evasion. Therefore, immune checkpoint inhibitors would enhance anticancer immunity [49]. We used the TIDE algorithm to predict clinical responses of immune checkpoint inhibitors as previously described [42, 50].

### Compounds therapeutic response prediction

CMap was used to predict the target therapeutic compounds using the top 1,000 differentially expressed genes [48]. Further, we used the R package “pRRophetic” to predict the half maximal inhibitory concentration (IC_50_) of chemotherapeutics obtained from the GDSC website in patients with LUAD [51].

### Independence of methylation-based model from patients’ clinical characteristics

To examine whether prognostic and recurrence models were independent variables as compared with other conventional clinical characteristics (age, gender, and TNM, T, N, and M stages) in patients with LUAD, univariate and multivariate Cox regression analyses were performed.

## Supplementary Material

Supplementary Figures

Supplementary Table 1
