# Supplementary material for: DNA methylation-based lung adenocarcinoma subtypes can predict prognosis, recurrence, and immunotherapeutic implications
Source: Aging (Albany NY). 2020 Nov 21;12(24):25275–93. doi: 10.18632/aging.104129 (PMC7803536; doi:10.18632/aging.104129)
Supplement: Supplementary Figures [file aging-12-104129-s001.pdf]

SUPPLEMENTARY FIGURES

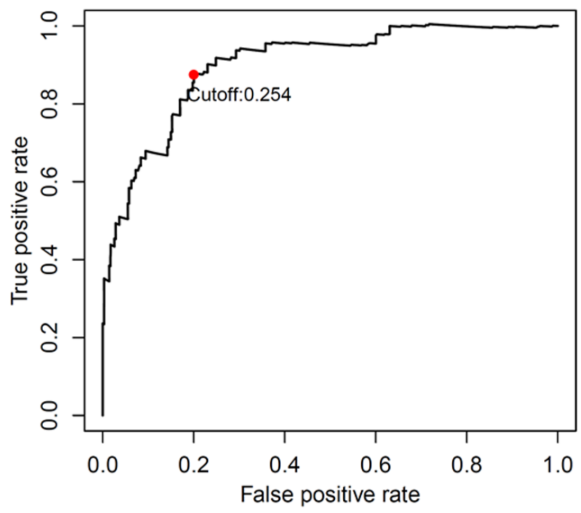

Supplementary Figure 1. Optimal cut–off value for the risk score.

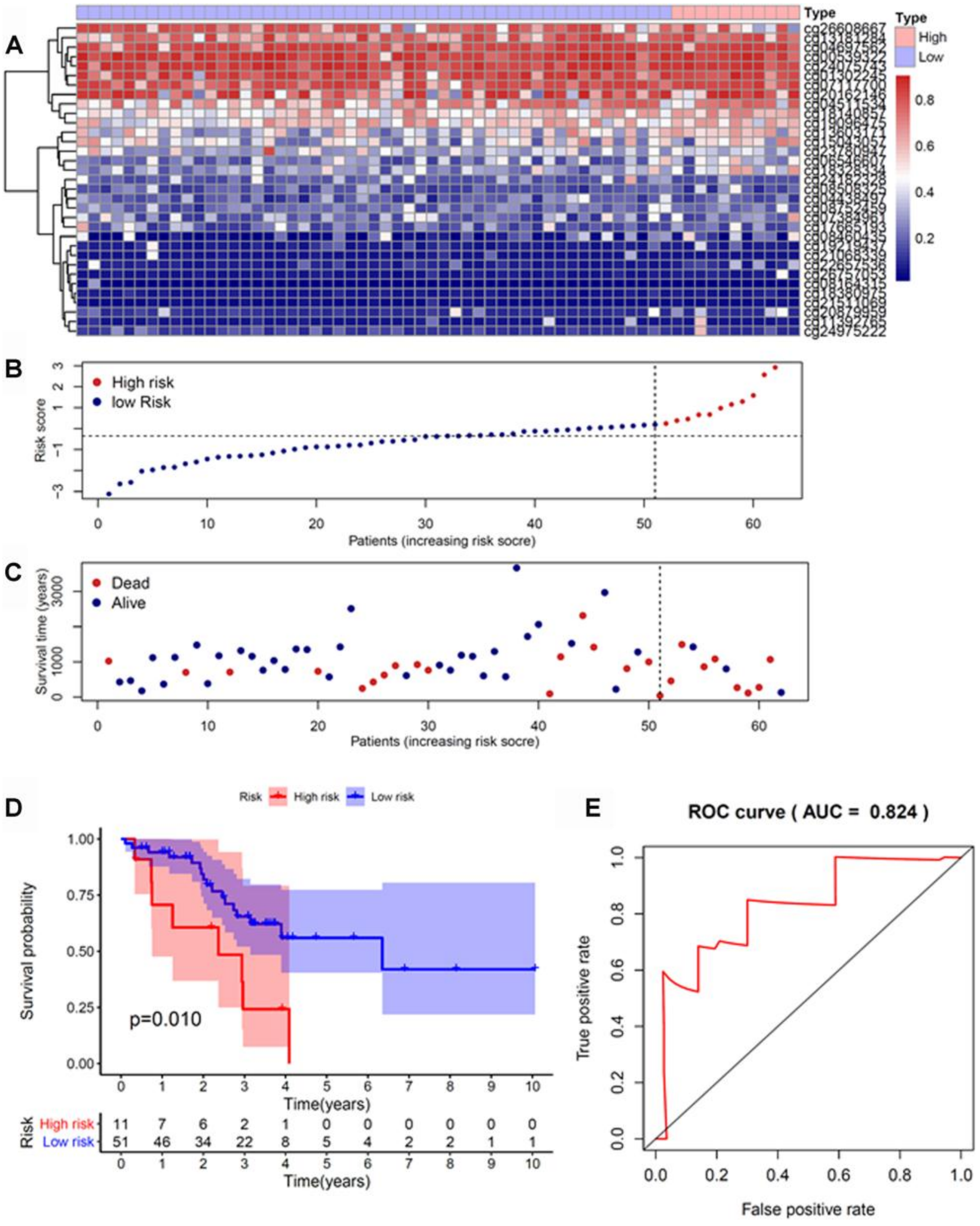

Supplementary Figure 2. Validation of the prognostic methylation model for patients with LUAD using the HM27K dataset. (**A**) The clustering analysis heatmap of methylation profile in DNA methylation signature sites. (**B**) Distribution of the DNA methylation-based risk score. (**C**) Vital status of patients in the high- and low-risk groups. (**D**) Kaplan–Meier survival curves of the relative overall survival of patients in the high- and low-risk groups. (**E**) Accuracy of the prognostic model in predicting survival time by ROC curve analysis. LUAD, lung adenocarcinoma; HM27K, HumanMethylation 27K; ROC, receiver operating characteristic.

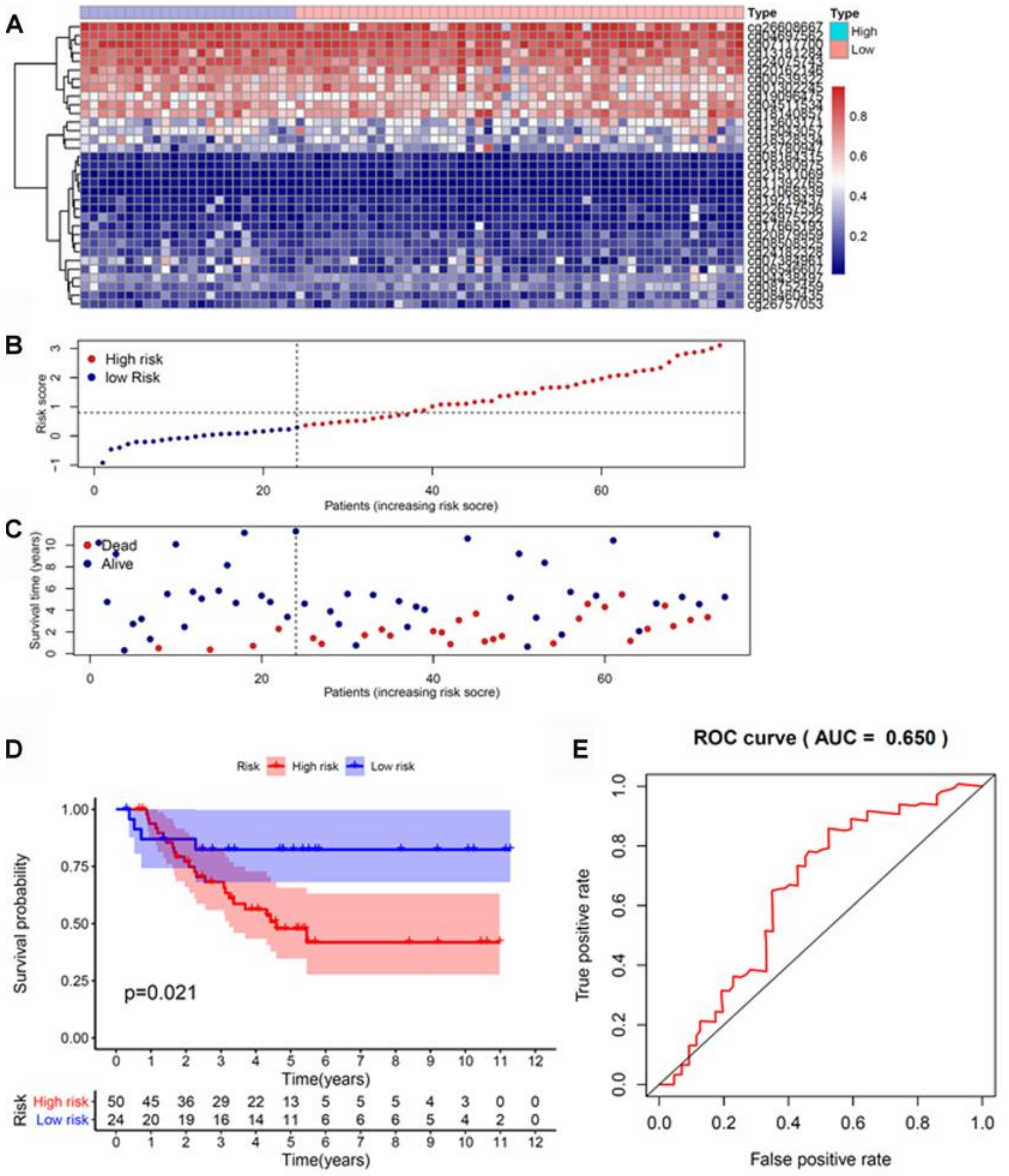

Supplementary Figure 3. Validation of the prognostic methylation model for patients with LUAD using the meta-GEO dataset. (**A**) The clustering analysis heatmap of methylation profile in DNA methylation signature sites. (**B**) The distribution of the DNA methylation-based risk score. (**C**) Vital status of patients in the high- and low-risk groups. (**D**) Kaplan–Meier survival curves of the relative overall survival of patients in high- and low-risk groups. (**E**) Accuracy of the prognostic model in predicting survival time by ROC curve analysis. LUAD, lung adenocarcinoma; GEO, Gene Expression Omnibus; ROC, receiver operating characteristic.

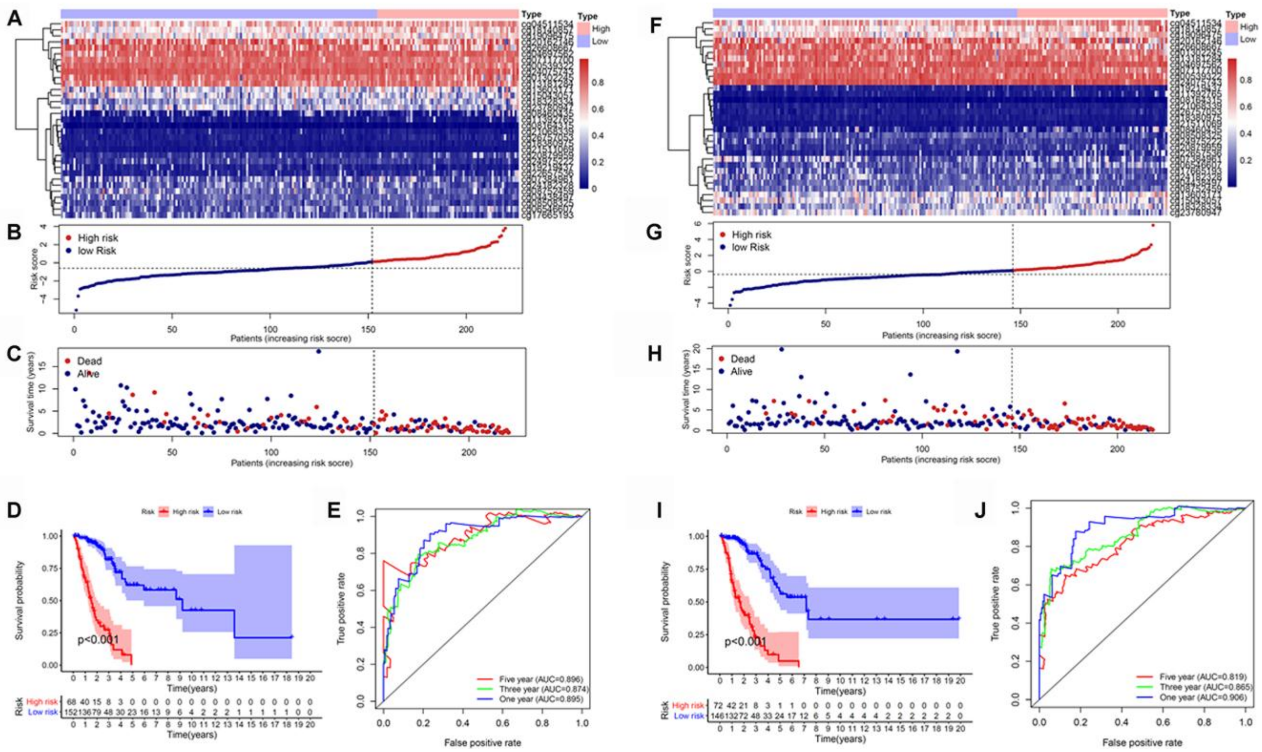

Supplementary Figure 4. Validation of the prognostic methylation model for patients with LUAD using TCGA internal validation. (**A**) The clustering analysis heatmap of methylation profile in the DNA methylation signature sites for the training set. (**B**) Distribution of the DNA methylation-based risk score for the training set. (**C**) Vital status of patients in the high- and low-risk groups for the training set. (**D**) Kaplan–Meier survival curves of the relative overall survival of patients in high- and low-risk groups for the training set. (**E**) Accuracy of the prognostic model in predicting survival time by ROC curve analysis for the training set. (**F**) The clustering analysis heatmap of methylation profile in DNA methylation signature sites for the testing set. (**G**) Distribution of the DNA methylation-based risk score for testing set. (**H**) Vital status of patients in the high- and low-risk groups for the testing set. (**I**) Kaplan–Meier survival curves of the relative overall survival of patients in the high- and low-risk groups for the testing set. (**J**) Accuracy of the prognostic model in predicting survival time by ROC curve analysis for the testing set. LUAD, lung adenocarcinoma; ROC, receiver operating characteristic; TCGA, The Cancer Genome Atlas.
