# Supplementary material for: DNA methylation-based lung adenocarcinoma subtypes can predict prognosis, recurrence, and immunotherapeutic implications
Source: Aging (Albany NY). 2020 Nov 21;12(24):25275–93. doi: 10.18632/aging.104129 (PMC7803536; doi:10.18632/aging.104129)
Supplement: Supplementary Table 1 [file aging-12-104129-s002.pdf]

SUPPLEMENTARY TABLE

Supplementary Table 1. Summary of patients' characteristics.

Characteristics	Training set		Testing set		*p*-Value
	Number	Percentage	Number	Percentage	
Age (years)					
<60	59	26.82	64	29.36	0.5823
≥60	157	71.36	148	67.89	
Not available	4	1.82	6	2.75	
Sex					
Female	116	52.73	114	52.29	1
Male	104	47.27	104	47.71	
Stage					
Stage I–II	171	77.73	173	79.36	0.9805
Stage III–IV	45	20.45	44	20.18	
Not available	4	1.82	1	0.46	
T					
T1–T2	190	86.36	192	88.07	0.6682
T3–T4	29	13.18	24	11.01	
Not available	1	0.45	2	0.92	
M					
M0	141	64.09	136	62.39	0.9909
M1	10	4.55	9	4.13	
Not available	69	31.36	73	33.49	
N					
N0	141	64.09	146	66.97	0.7056
N1–N3	73	33.18	69	31.65	
Not available	6	2.73	3	1.38	
